# Transgenic Strategies to Study Podocyte Loss and Regeneration

**DOI:** 10.1155/2015/678347

**Published:** 2015-05-18

**Authors:** Duccio Lombardi, Laura Lasagni

**Affiliations:** ^1^Excellence Centre for Research, Transfer and High Education for the Development of DE NOVO Therapies (DENOTHE), University of Florence, 50139 Florence, Italy; ^2^Department of Clinical and Experimental Medicine, University of Florence, 50139 Florence, Italy

## Abstract

Podocyte death and regeneration are major topics in kidney research but remain controversial. Data obtained in humans demonstrate the existence of cells sited along Bowman's capsule that behave as podocyte progenitors *in vitro* and in *in vivo* mouse models of podocyte injury xenotrasplanted with this human-derived population. However, this podocyte reservoir still remains elusive in murine models, where it could be more easily studied. Transgenic models can be a powerful tool to identify this population and to better understand its dynamics and hierarchies in both physiological and pathological conditions. Indeed, exploiting transgenic approaches allows detecting, at the single cell level, movements, cell death, and replacement. Moreover, through lineage tracing it is now possible to identify specific population increase and to point out clonal expansions during or after the regenerative processes. However, applying transgenic strategies to study glomerular regeneration requires the search of markers to unequivocally identify this progenitor population. Achieving this aim would lead to a deep comprehension of the biological processes that underlie glomerular regeneration and clarify how different cell pools interface during this phase. Here we discuss strategies that have been used and new approaches in transgenic models finalized to study podocyte loss and subsequent replacement.

## 1. Introduction

A crucial element of the nephron filtration barrier is a specialized epithelial cell known as the podocyte. A vast majority of renal diseases start with the dysfunction or loss of podocytes, resulting in proteinuria that leads to nephron degeneration and to kidney failure. No clinical methods are actually available to heal podocyte damage, and the progressive loss of nephrons leads to failure of kidney function and, finally, to end-stage renal disease (ESRD). Interestingly, several studies have suggested that podocyte stem/progenitor cells exist in humans providing a possible explanation for the podocyte regeneration observed in several clinical and experimental situations [1–3]. The existence of a progenitor reservoir appointed to podocyte replacement has been clearly demonstrated in other vertebrates like zebrafish [4–6]. On the contrary, there are still conflicting opinions on the notion of podocyte regeneration and supporting the identity of the putative progenitor cells in the mouse. Given the consequences of podocyte health for overall renal function, to definitely elucidate the mechanism of podocyte regeneration in mammals is of primary relevance. To this aim, the choice of the most adequate model system and the correct interpretation of the data obtained from a specific model are the main points that researchers must take into consideration. In the last years, new methods that enable us to provide extremely accurate data have been searched and developed. In particular, transgenic mouse models offer important tools for research in the field of podocyte regeneration. For instance, podocyte loss is the trigger stimulus in the chain of events that lead to podocyte regeneration, and now temporally and spatially regulated genetic systems for podocytes ablation are available and allow researchers to strictly control podocyte death. In addition, inducible mouse models of lineage tracing enable us to follow the events of regeneration in real-time by labeling a specific cell type and tracking its fate. In this review, we provide an introduction to transgenic mouse models, an overview of transgenic mouse systems to induce podocyte-specific cell death, discuss how transgenic mouse studies have been used to evaluate podocyte regeneration, and how new transgenic strategies might help researchers in definitively addressing mechanisms of podocyte regeneration.

## 2. Introduction to Transgenic Models

In the last decades, transgenic animals have been intensively developed and used in order to understand how a determinate gene deletion in a specific cell type correlates with a particular phenotype, which is the role of a certain gene in organ development, or which is the response of organ-specific stem cells to a particular kind of injury [7–9]. Moreover, in transgenic models it is possible to mark certain cell populations, study them at different time points, follow a specific cell pool to understand its role during regeneration, and exploit other benefits that will be discussed in this review.

**Figure 1:**
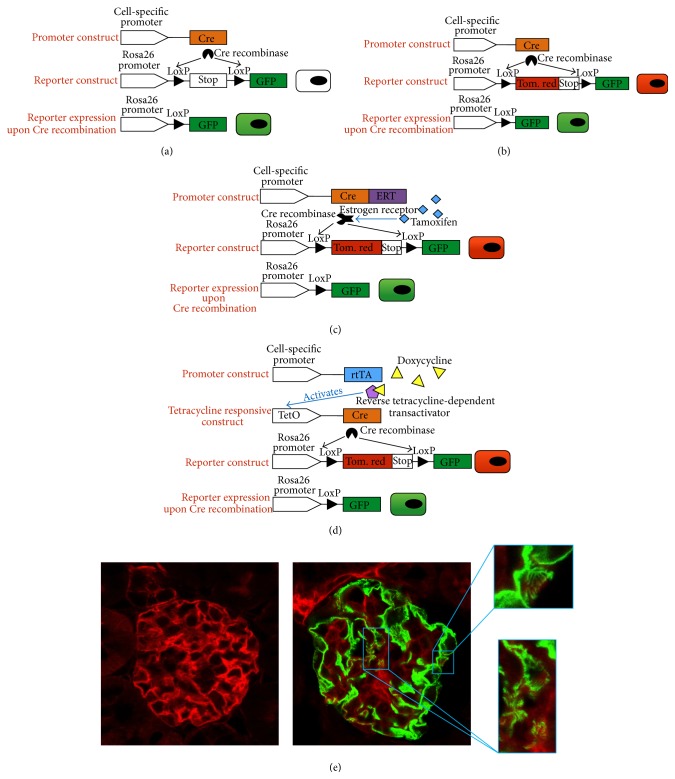
Schematic representation of the principal transgenic solutions. Each line represents a mouse strain, which is crossed with other transgenic strains in order to obtain double or triple transgenic animals. (a) Cell-specific promoter directly controls Cre activity creating a constitutive model. When the promoter gets activated Cre recombinase will be expressed and will gain access to LoxP sites. By removing the LoxP-floxed Stop cassette, the recombinatorial event will lead to GFP expression. (b)* mT*/*mG* (membrane-Tomato/membrane-Green) reporter construct in a constitutive scheme. The cell-specific promoter, in this setting, will change desired population color from ubiquitously expressed tomato red protein to GFP, evidencing cells that had recombinatorial activity. (c) Inducible model based on a fused form of Cre recombinase and estrogen receptor (ERT) proteins. If the chosen promoter is activated this fused protein will always be transcribed. Only following Tamoxifen treatment the ERT will bind the inductor and enter the nucleus, where Cre recombinase will act on the LoxP site of the reporter transgene* mT*/*mG*. (d) Inducible model based on reverse Tetracycline-controlled transactivator (rtTA) protein, which is continuously created by the activated specific promoter. When bound to Tetracycline (Doxycycline form), the transcriptional factor is able to activate Cre transcription controlled by TetO operator sequences. (e) Imaged glomeruli in a podocyte-driven* mT*/*mG* setting prior to (left panel) and following (right panel) induction. The strong fluorescence signal of this reporter line enables the visualization of podocyte primary foot processes (right enlarged box).

In the past years, a big setback related to the creation of transgenic animals has been the random genomic integration of the foreign DNA (called transgene). This randomness could result in loss of functionality of endogenous genes or in the disconnection of a gene from the elements that control its expression, leading to genetic deregulation that could alter the physiological state of a cell. On the contrary, transgenes may be inserted in chromosome sites that have been silenced, thus resulting in low or complete loss of transgene expression [7]. Nowadays it is possible to insert the exogenous DNA in a specific* locus* of the animal genome thanks to a process based on homologous recombination, called gene targeting. By gene targeting it is thus possible to introduce, in the animal genome, reporter transgenes which encode for markers that can be used to identify, visualize, and track a desired cell type, or different cellular populations, in pathological as well as in physiological organ contexts. These reporters generally codify for fluorescent proteins and are commonly inserted under the Rosa26 locus by homologous recombination. To label a single cell or to tag a specific population with a fluorophore, without confusing this population with others, it is essential that the genetic mechanisms that lead to the reporter expression are controlled in a cell-specific manner. To achieve this aim, cell-specific promoters, generally genes expressed only by the population of interest, are engineered in order to induce the expression of a recombinase enzyme. This enzyme, in turn, will modify the DNA sequence of the reporter gene to “unlock it” and make it expressible. The most common system used for genetic recombination is the Cre recombinase-LoxP system, in which Cre recombinase, expressed under the control of a tissue- or cell-specific promoter, specifically activates the reporter expression by excising the Stop sequence flanked by two LoxP sites (Figure 1(a)). If the Cre recombinase recognizes two LoxP sites oriented in a head-to-tail manner, it will remove the interposed (called “floxed”) sequence, while rejoining the ends together. Otherwise, if the LoxP sites are oriented in a head-to-head manner, the interposed sequence will be simply inverted by the enzyme [10–13]. Cre recombinase is widely employed to tag stem cell populations of various organs and to discern them from other cell pools [8, 14, 15]. Notably, its use combined with the* mT*/*mG *(*membrane-Tomato*/*membrane-Green*) reporter transgene enables us to identify cells that have undergone genetic recombination. Indeed, this system allows us to track a switch from a constitutive expression of the red fluorescent protein tomato red, encoded in a cassette floxed by two LoxP sites, to the expression of the downstream GFP (green fluorescent protein) gene (Figure 1(b)).

The use of reporter transgenes has gained a pivotal role and an increasing relevance in a very important tool for* in vivo* experiments focused on regeneration: lineage tracing analysis. Lineage tracing is defined as the possibility to tag a single desired cell with a reporter and to identify all the progeny derived from this cell. Since the irreversible DNA modification that leads to the expression of the reporter is inherited by all the daughter cells, it is possible to quantify the progeny of a definite founder cell and follow its daughters' behavior in physiologic as well as pathologic conditions [10]. However, lineage tracing analysis is based on a further evolution of transgenic models, the so-called inducible models, which enable a time-based regulation of the Cre recombinase activity. Indeed, in the conditional approaches described above, genetic tracing labels all the cells that, in any moment of their life, activated the cell-specific promoter driving Cre expression. This* in vivo* genetic fate mapping is frequently confused with lineage tracing analysis and can give controversial results. For instance, upregulation or transient activation of the chosen cell-specific promoters in a different or unexpected cell population can lead to erroneous experimental result interpretation. In inducible models, cell-specific promoters induce Cre activity only as a consequence of the administration of exogenous molecules that act as inductors, as in the Tamoxifen-regulated system (CreERT) or in the Tetracycline-regulated system (Figures 1(c) and 1(d), resp.). Thus, the desired cell pool is tagged only during the administration window of the inductor molecule, making it impossible for a cell to undergo genetic recombination upon withdrawal of the molecule, even if promoter-positive.

**Figure 2:**
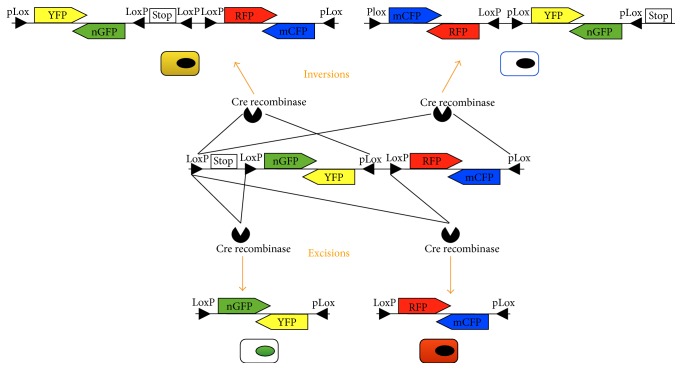
Schematic overview of the Confetti reporter system. In the center, the whole construct. On the bottom, excision eliminates reporter gene thus turning on one cell color. On the top, inversions rely on pLox sites that are used to invert the construct favoring other colors (Cre recombinase inverts segments floxed between two LoxP sites oriented in a head-to-head fashion). Both inverted and excised fragments can continue to invert as long as there is Cre activity, since LoxP sites are excised, while pLox sites are not. Consequently, an inducible model might be best suitable to stabilize cell color following inductor molecule removal. All recombinatorial events that do not involve the LoxP site upstream to the Stop cassette would result in no color acquisition by the cell.

The introduction, in the last years, of multicolor reporter constructs has increased the data output from tracing analysis. Of particular interest, Livet et al. developed a system, called Confetti [13], that was extensively used to better understand stem cells dynamics during regeneration, to discriminate different cell types contribution in the repair phase and to evidence clonal expansion [14, 16, 17]. This reporter enables the expression of one out of four fluorescent proteins, in a stochastic manner, by using alternating incompatible LoxP variants (LoxP and pLox) and by linking reporter genes* in tandem* with opposite DNA orientation in between these LoxP sites. These variants give rise to mutually exclusive excision of fluorescent reporters, thus inducing the cell to randomly acquire one out of four colors [13] (Figure 2).

The Confetti reporter not only enables the examination of the individual behavior of multiple stem cells in a single niche but also allows us to study how different positional effects in the niche can affect differentiation of stem or progenitor cells [16, 17]. Moreover, the randomness of the recombination events and the possibility to tag cells with different fluorophores allow us to easily visualize clonal expansion of single cells that will appear as continuous clusters of cells of the same color. Cellular hierarchies can thereby be resolved with high definition, without confounding progenitors with cells under transient state changes (i.e., dedifferentiation), which complicate accurate cellular identification by antibody-based methods.

All these discussions evidenced how much transgenic strategies would be useful in deciphering regenerative routes. In the following sections, we will focus on transgenic tactics to induce podocyte loss and, consequently, evaluate their* de novo* generation.

## 3. Podocyte Ablation: Transgenic Strategies to Induce Podocyte Loss

The major question related to glomerular regeneration is centered on the podocyte, a cell with an extremely complex cytoskeleton that composes the glomerular filtration barrier and which is the target of a broad range of chemical and physical insults. Indeed, podocytes react to immune- and nonimmune-mediated injury with rearrangement of the complex actin cytoskeleton, spreading of the foot processes along the GBM (glomerular basement membrane), loss of filtration slits, and apical redistribution of slit diaphragm proteins. Consequently, a progressive reduction in podocyte number from podocyte death and/or detachment is observed and leads to the development of glomerulosclerosis, the pathological basis of chronic renal failure. In human, evidence demonstrated that a population of cells localized in the parietal epithelium of Bowman's capsule possesses characteristics of stem/progenitor cells. This population can be expanded as clones in culture and can differentiate into podocyte both* in vitro* and* in vivo* following intravenous administration in a SCID (severe combined immunodeficiency) mice model affected by adriamycin nephropathy (AN), the experimental analogous of human focal segmental glomerulosclerosis (FSGS) [1, 2]. However, the existence of this population in mouse is still an open question whose answer is of paramount importance in regenerative nephrology research. Indeed, a greater understanding of podocyte biology and of the regenerative role of renal progenitors may provide novel therapeutics to treat glomerular disease. Any attempt to identify a progenitor pool able to regenerate lost podocytes relies on experimental procedures to induce podocyte loss. In humans, podocyte damage is the starting event leading to FSGS, a disease histologically characterized by the presence of sclerosis of part of the glomerular capillaries in a minority of glomeruli. Clinically, podocyte loss results in proteinuria, hypoalbuminemia, hypercholesterolemia, and peripheral edema, that is, nephrotic syndrome. FSGS is not a single disease but a lesion as a result of different pathophysiologies that cannot be recapitulated by a single animal model. Thus, different animal models of both primary and secondary FSGS, with specific pros and cons, have been developed to mimic the clinical pathological features of human FSGS [18, 19]. The choice of the model most appropriate to the experimental purpose must be guided by the question that the researcher is trying to solve. In this review we will focus our attention on models of FSGS obtained using transgenic mice. However, we will provide also a brief description of the two most widely used nontransgenic models: the remnant kidney and adriamycin nephropathy. In the remnant kidney model, 4/6 or 5/6 of the total renal mass is surgically removed by unilateral nephrectomy coupled to ligation of renal artery branches or polectomies in the contralateral kidney. These surgical procedures lead to hypertension, renal damage, and FSGS (which is more severe in the 5/6 model). The remnant kidney model is mainly used in rats. Indeed, the anatomic distribution of the renal artery branches makes it difficult to achieve reproducible 5/6 nephrectomy in mouse; moreover, while 5/6 nephrectomy may be sufficient to evoke development of FSGS in 129/Sv male mice within 1-2 months, most mouse strains, including the C57Bl/6 strain, are resistant to this procedure and do not develop FSGS [20, 21]. In C57Bl/6 mice, it is necessary to treat 5/6 nephrectomized animals with deoxycorticosterone acetate (DOCA) and a high salt diet to induce FSGS. These procedures result in hypertension and nephrosclerosis (an endocrine hypertension model). However, like for the remnant kidney model, the degree of hypertension and hypertensive renal lesions is markedly different between various mouse strains commonly used in research, with 129/Sv strain more susceptible than C57Bl/6 [22]. Surprisingly, in this mouse model, the amount of albumin excretion did not correspond to the degree of glomerular damage and therefore albuminuria is not a good predictor for the degree of glomerular scarring, making the screening of kidney injury for treated mice more challenging [22].

FSGS can also be induced by treating animals with podocyte-toxic drugs, such as adriamycin (Pfizer, Sydney, Australia) (doxorubicin). After the initial toxic injury, mice develop an immune-mediated chronic proteinuric renal disease. The clinic pathological features of AN are nephrotic syndrome, focal glomerulosclerosis, tubular injury, and interstitial compartment expansion with infiltration of mononuclear cells that are composed largely of macrophages and T cells. In mice, AN is associated with acceptable mortality (less than 5%) and morbidity (weight loss) [18]. Nevertheless, several critical points must be taken into consideration when performing adriamycin nephropathy experiments. As first issue, the identification of the optimal regimen of adriamycin administration requires great effort because of the variability between species, strain, gender, and age of the animals and even differences between animals of the same litter. Most rat species are completely sensitive to the renal effects of adriamycin. In male Wistar rats, the dose of adriamycin ranges between 1.5 and 7.5 mg/kg. Instead, male BALB/c mice require 9.8–10.4 mg/kg, [23] while male SCID mice developed on a BALB/c background require only 5.3 mg/kg [24]. C57Bl/6 mice are highly resistant to adriamycin-induced renal injury, but renal injury may be inducible at higher doses (13–25 mg/kg) than those required for BALB/c mice [25–27]. In C57Bl/6-based models, while most studies use a single injection, regimens using multiple injections (i.e., 2 mg/kg × 2 in 20 days, 1 mg/kg/day × 7 days, and 2.5 mg/kg × 6 in 14 days) have also been reported. The choice of the effective dose is further complicated by the variability among drug sources and batches. To overcome these problems, dose-finding tests are usually necessary to ascertain the exact dose necessary to induce the pathological changes required by the investigator. Finally, adriamycin has also effects that are not specific to the kidney such as myelotoxicity, hepatotoxicity, cardiomyopathy, and neurotoxicity as evidenced by lack of animal coordination [18, 28].

Although AN and remnant kidney models both resemble human FSGS, adriamycin nephropathy, which induces podocyte oxidative stress, fostered the concept of primary podocyte injury in the pathogenesis of foot process effacement and glomerulosclerosis. By contrast, renal ablation models pointed to glomerular hypertension (elevated glomerular capillary pressures and flow rates) as the primary pathophysiologic process in the course of adaptive responses to reduced number of functioning nephrons, in turn causing secondary podocyte injury [29, 30].

Advances in transgenic and gene-targeting technologies have elucidated the function of individual genes in glomerular health and disease. Several animal models are now available, in which a transgene insertion or the modification of a gene in podocytes leads to a more or less severe albuminuria, sometimes of nephrotic range, and focal glomerulosclerosis [31–34]. However, conditional cell-targeted ablation constitutes the most promising genetic tool to analyze cell lineage relationships, the role of specific cells during embryogenesis, or physiological processes. Moreover, the ability to control temporally and spatially the tissue damage and to genetically remove a specific cell population has important applications for regeneration studies. The ideal genetic cell ablation tool must be (1) spatially controllable and strictly confined to the target cell population, (2) temporally inducible, (3) germline transmissible, and (4) reversible. In this paragraph we will focus our attention on three of the most commonly used transgenic models in which genetically controlled cell removal ensures the highest precision and consistency in studying the consequences of podocyte damage. As a consequence, studies on mechanisms of podocyte regeneration can be more accurately performed.

*Thy-1.1 Model*. In their endeavor to study the function of the Thy-1 antigen, Kollias et al. [35] generated transgenic mice that ectopically express a mouse-human chimeric Thy-1.1 antigen. Some of these transgenic mice expressed the Thy-1.1 antigen specifically on podocytes, slowly and spontaneously developing albuminuria (from week 7 onward) and focal glomerulosclerosis [35, 36]. After podocyte-specific strain isolation, the model has been extensively used in experimental settings based on the anti-Thy-1.1 monoclonal antibody (mAb) injection. mAb administration induced an acute albuminuria in nonalbuminuric, 5-week-old Thy-1.1 transgenic mice, while the same procedure did not cause albuminuria in nontransgenic mice [36]. Smeets et al. described in detail the dose-dependent effects of anti-Thy-1.1 mAb treatment [37]: high dose injection (1000 mg) induces an albuminuria within 10 min that persisted over a 3-week period. With lower doses (100 *μ*g) of anti-Thy-1.1 mAb, on the contrary, the developed albuminuria rapidly dropped and returned to baseline levels within 7 days. Glomeruli became increasingly ischemic with no sign of podocytic hypertrophy or swelling. Electron microscopy revealed reduction in number of foot processes and decrease in diameter of slit pores already 10 minutes after mAb injection, while no large gaps between podocytes or podocyte detachment were observed [37]. In this respect, this model closely resembles human nephropathies such as minimal change disease or FSGS. In contrast to adriamycin that causes severe podocytic injury leading to necrosis and detachment of the podocytes from the GBM, in this model, the podocyte injury is relatively small, with no detachment detectable in the first 24 h after mAb administration. Moreover, in AN the level of proteinuria correlates with the degree of podocyte detachment, while in the anti-Thy-1.1 model podocyte injury is relatively minor despite massive proteinuria. Actually no Rosa26 targeted Thy-1.1 mice line exists that can be exploited with the Cre-LoxP strategy, thus hampering the possibility to specifically control transgene expression by using constitutive or inducible podocyte-specific promoters.

*NEP25 Model*. This model relies on an immunotoxin-mediated cell targeting technology. LMB2 is a chimeric protein composed of the Fv portion of an anti-Tac (human CD25) antibody and PE38, a mutant form of Pseudomonas exotoxin that contains the translocation and ADP ribosylation domains. LMB2 has been used to ablate specific cell types in transgenic mice that express hCD25 in the target cells [38, 39]. Transgenic mouse lines that express hCD25 selectively in podocytes, as the one directed by the nephrin promoter, have been successively developed [40]. Two to three days after LMB2 injection these mice developed nonselective proteinuria, hypoproteinemia, ascites, edema, and renal failure. Because of severe edema and/or renal failure, most transgenic mice died at a time inversely related to the dose of LMB2 injected. With 0.625 ng/g body weight of the toxin, the majority of the transgenic mice showed mild and transient ascites and survived for more than 28 days. Proteinuria peaked at the seventh day and, thereafter, gradually decreased with time, returning nearly to the normal range within 28 days [40]. Light microscopy, transmission and scanning electron microscopy, and immunostaining for podocyte marker proteins demonstrated damage and progressive loss of podocytes, but transferase-mediated dUTP nick-end labeling detected only rare apoptosis in the glomerulus indicating that apoptosis is not the major mechanism of the podocyte loss [41]. In addition to podocytes, glomerular endothelial cells, mesangial cells, parietal epithelial cells (PECs), and, later, tubular cells became damaged. Mice that survived for more than 3 weeks developed segmental or global glomerular sclerosis. It is interesting that, 4 weeks after LMB2 injection of 0.625 ng/g per body weight, the average scores of epithelial injury and mesangial changes were better than those measured at 3 weeks after the damage onset [40]. In addition, urinary protein was almost in normal range 4 weeks after the toxin administration. These suggest that glomeruli that are only mildly injured by LMB2 have the ability to recover. The advantages of this model are that transgenic mice are completely normal unless they receive LMB2 treatment, facilitating colony maintenance and reproduction. Moreover, the damage severity is dose-dependent and controlled by LMB2 quantity injection.

**Figure 3:**
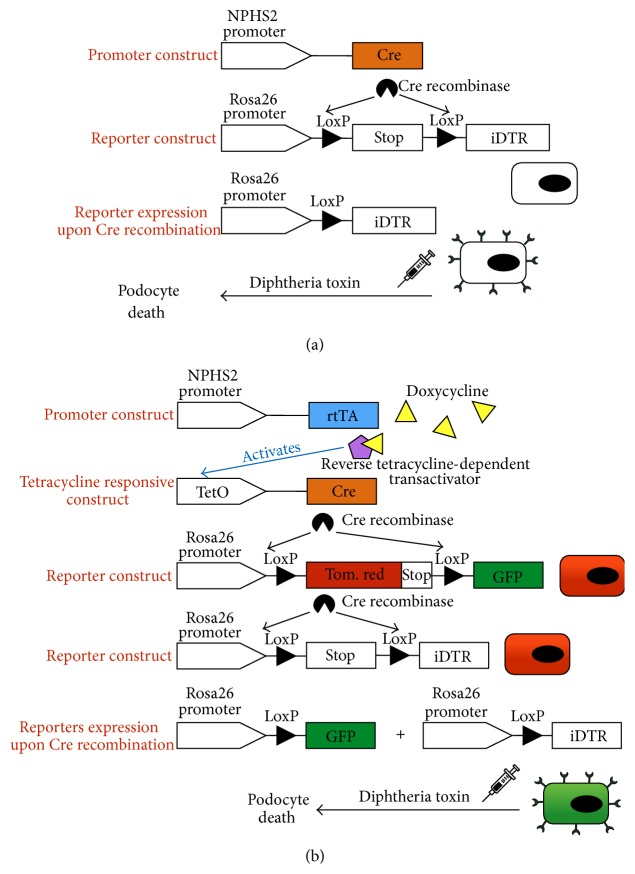
DTR models and strategies. (a) Schematic representation of a floxed Stop cassette followed by DTR transgene targeted under the Rosa26 promoter. The reporter transgene, activated upon Cre recombinase activity induced by podocin promoter activation, forces podocytes to express the DT receptor. The operator can thus easily induce podocyte cell death in a time- and dose-controlled manner. (b) Quadruple transgenic hNPHS2.rtTa; TetO.Cre;* mT*/*mG*; iDTR, in which podocytes can be prompted to turn from red to green while expressing the DTR. Once induced, the authors provoked podocyte death through DT injection [52].

*DTR Model*. The A chain of diphtheria toxin (DTA) catalyzes the ADP-ribosylation of the eukaryotic elongation factor 2, resulting in inhibition of translation, which finally leads to cell death [42, 43]. However, rodents are resistant to DTA because rodent heparin-binding epidermal growth factor-like growth factor does not bind the toxin. On the contrary, the forced expression of the human DT receptor (DTR) in a target cell population is able to render mouse cells susceptible to the toxin effects, triggering cell death [44]. Alternatively, it is possible to induce endogenous expression of DTA in the target cell, as proposed by Breitman et al. [45] and Palmiter et al. [46]. Recently, a conditional mouse line in which the expression of DTA can be controlled by Cre recombinase activity has been generated, thus allowing widespread ablation of different cell types by the use of tissue specific Cre-lines. Lately, Brockschnieder et al. described a mouse line in which a LoxP conditional DTA allele was introduced into the ubiquitously expressed Rosa26 locus [47]. Wharram et al. successfully used this strategy to study the pathological consequences of podocyte injury in adult rats [48], evidencing mesangial matrix expansion, formation of synechiae, and development of sclerosis areas with collapse of glomerular capillaries. Analogously, Jia et al. described a mouse podocyte ablation model in which the podocin (NPHS2) promoter directs Cre-mediated DTA176 (an attenuated DTA gene) production and subsequent podocyte ablation during nephrogenesis [49]. What renders this model so interesting is that* in vitro* studies suggest that internalization of as little as a single molecule causes a cell to die [50]. Recently, a Cre-inducible transgenic mouse strain, in which a LoxP-flanked Stop cassette and the simian diphtheria toxin receptor (iDTR) gene were targeted under the Rosa26 locus of mouse genome, has been generated (iDTR mouse) [51] (Figure 3(a)). iDTR mice can be crossed with any kind of Cre recombinase-expressing mice in which the enzyme is active only in a specific cell pool due to cell-specific promoter activation. Wanner et al. [52] crossed the iDTR strain to hNPHS2.rtTA, TetO.Cre, and* mT*/*mG* mice to specifically target podocyte ablation, in a dose-dependent manner, and determine the influence of acute podocyte loss on podocyte regeneration (Figure 3(b)). High doses (25–100 ng/g body weight) of DT resulted in massive albuminuria and loss of nearly all podocytes, whereas lower doses (2 and 5 ng/g body weight) were sufficient to cause a net loss of approximately 12% of all podocytes after 4 weeks, without imposing any persistent gross damage to the tissue. This injury led to a transient increase in albuminuria, which gradually decreased over 28 days [52].

The strength of the NEP25 and DTR models is that, by targeting specific cell populations, they show no obvious gender differences, as what happens in AN-based models, and that pathological and morphological changes can be modulated simply by adjusting the drug dose. The intrinsic limit of these models is that they cause specific but acute podocyte ablation, which is optimal to study podocyte* de novo* creation, but it does not closely resemble the vast majority of human podocyte-related pathologies, in which podocyte loss is a long-standing and progressive event.

In conclusion, several good models that recapitulate FSGS are available; nevertheless, choosing the correct model to address specific hypothesis is the essential prerequisite for the success of research.

## 4. Podocyte Regeneration: Transgenic Tactics to Identify Progenitor Pool for Podocyte Replacement

Transgenic models have been widely employed, in the last ten years, to identify stem or progenitor populations present in the glomerular compartment and to understand their role in podocyte turnover in both homeostasis and pathological states. One of the first groups that used genetic strategies to identify podocyte reservoir has been Moeller's group that, in 2009, using a fused form of human and rabbit podocalyxin as promoter, tagged parietal epithelial cells of Bowman's capsule [53], already described in humans as podocyte progenitors [2]. By employing this inducible mouse model, the authors were able to specifically label mature PECs with *β*-gal, allowing the direct visualization of this compartment for the first time in rodents [53]. This intriguing strategy that later was found to be essential for the discovery of PEC pathological participation in glomerular sclerotic lesions and in crescent formation [54, 55] allowed us to show that PECs labeled at postnatal day 5 migrated to the glomerular tuft and differentiated into podocytes, providing evidence, confirmed also by Wanner et al., that some podocytes are recruited from PECs at least in juvenile mice [52, 53]. In a more recent paper, the same group confirmed that, during adolescence, generation of podocytes occurs from a subpopulation of PECs that the authors called “podocyte reserve,” that is, cells already committed to become podocytes, which shows a transitional phenotype and can be found in Bowman's capsule only in juvenile mice and not in adult mice [56]. Using the same lineage tracing model, podocyte generation from PECs could not be detected in aging mice or in models of glomerular hypertrophy (5/6 nephrectomy and DOCA-salt model) [56]. It is reported that stem/progenitor cells participate not only in regenerative response to acute injury but also in the general maintenance function of replacing cells that are lost during physiologic organ activity, that is, homeostasis. However, some tissues such as muscle and brain exhibit limited turnover. The finding on aging reported by Berger et al. [56] and confirmed also by Wanner et al. [52] suggests that this is true also for the kidney: podocytes have long lifespan and are rarely replaced. It is possible that the aging process in the glomerulus is accompanied by subtle and gradual effects that are compensated during time mostly by nephron surplus [57] and podocyte hypertrophy [57, 58] and thus do not involve the putative progenitor compartment. Interestingly, even in the liver, a tissue with a dramatic regenerative capacity, stem cells are not used during homeostasis but are called into service only upon injury [59, 60]. However, Berger et al. fail to evidence a role for PECs even after injury using two models of glomerular hypertrophy. Indeed, they observed that PECs participate only in the formation of sclerotic lesions, whereas hypertrophied glomeruli did not show migration of genetically tagged PECs to the glomerular tuft, thus arguing against the role of PEC in podocyte regeneration [56]. However, a questionable point in the papers from Appel et al. [53] and from Berger et al. [56] remains the promoter used to tag putative progenitors: can the fused form of human and rabbit podocalyxin genes be used as a promoter to correctly identify progenitor cells?

In a recent paper, Hackl et al. [61], employed a constitutive transgenic mice model controlled by the rat phosphoenolpyruvate carboxykinase promoter (PEPCK), crossed with a GFP reporter strain, to genetically tag PECs. The authors, using a unilateral ureteral obstruction (UUO) disease model, evidenced rare GFP-positive PECs that migrated from Bowman's capsule vascular pole to the glomerular tuft. Nevertheless, this constitutive model does not allow us to unequivocally identify the progenitor pool because of the nonspecificity of the used promoters, which did not mark all PECs, while tagging also other pools [61] with a high grade of recombination in S1–S3 proximal tubular cells [62].

Sakamoto et al. [63] used a constitutive transgenic mouse expressing *β*-gal and human CD25 under the control of the nephrin promoter, allowing tagging of podocytes as to induce their specific ablation by LMB2 injection. Following podocyte ablation, the authors investigated phenotypic transition from podocyte to PECs or* vice versa* by double staining for podocyte or PEC markers. The conclusion is that podocytes undergo epithelial phenotypic transition into PEC [63]. However, by this transgenic tactic, they performed a genetic fate mapping of podocyte pool and are not* tracing the podocyte lineage cell* since any cell that might have expressed nephrin, at any moment and even for a very short period, would result as *β*-gal^+^. Thus, *β*-gal^+^ cells could result not only from nephrin expression in fully differentiated podocytes but also from progenitors starting to acquire this podocyte markers upon differentiation into podocyte lineage, as already reported in human [2] and rodents [53] studies of two different groups. This is further corroborated by the findings that, in the paper [63], claudin^+^ WT1^+^ cells were reported to mainly localize at the vascular pole, sustaining the idea of PEC differentiation to podocyte while migrating to cover the tuft along the denuded glomerular basement membrane. Furthermore, identification of cells by immunostaining for specific protein markers can lead to misinterpretation of results due to upregulation of markers as consequence of damage or as response to injury, also in more than one particular cell type.

A different hypothesis related to podocyte regeneration has been recently formulated by Pippin et al. who lineage-traced the cells of the juxtaglomerular apparatus by engineering the mouse genome with a modified and inducible form, of the renin gene [64]. The authors were thus able to show that renin-positive cells possess regenerative capacities because of their ability to substitute, in an experimental model of FSGS, not only lost podocytes but also PECs. This suggests that cells of renin lineage may have the capacity to serve as upstream progenitors for both PECs and podocytes [64]. However, this population localizes in the preglomerular vascular wall, and it is actually unclear how it would cross the parietal and the glomerular basement membrane to gain access to the glomerular compartment [65]. Moreover, the number of podocytes generated by renin expressing cells appeared extremely low and its relationship with the outcome of the disease has not been definitively proven. A subsequent study of the same group focused on the role of renin-positive cells in aging nephropathy [66], which is characterized by progressive podocyte loss and consequent focal and global glomerulosclerosis [67, 68]. In the paper, the authors used a constitutive model in which the renin gene drives the recombination that leads to ZsGreen fluorophore expression. Thanks to this genetic fate mapping, it was proved that, at 1 year of age, the intraglomerular compartment exhibited an increment in the number of cells of renin lineage, most of which expressed markers exclusively associated with differentiated podocytes (as podocin and synaptopodin) [66]. However, in this genetic fate mapping strategy based on a constitutive model is not possible to understand if the renin-positive pool can substitute lost podocytes, since any cell (and also podocytes [69, 70]) that had expressed renin, in any moment of the animal life, would be tagged by the reporter. The study by Starke et al. further challenged the involvement of cells of renin lineage in podocyte turnover with an inducible model that enabled tagging of the renin^+^ population with *β*-gal reporter transgene [71]. By setting up a mesangial injury in this transgenic model the authors proved that renin descendant cells never colocalized with podocyte or PEC markers, denying their role as podocyte or even PEC reservoir, evidencing instead a prominent role in intraglomerular mesangial cell substitution [71]. Thus, controversial findings in the literature still leave the role of renin-positive cells as podocyte progenitors undefined.

A recent and very elegant paper by Wanner et al. demonstrated how transgenic animals can be used to study glomerular regeneration and podocyte replacement in aging nephropathy, in a unilateral nephrectomy model and following acute podocyte loss [52]. To study podocyte regeneration the authors induced a severe podocyte depletion thanks to the mentioned transgenic tactics of specific ablation of cells engineered to express the iDTR transgene. To this aim, they created a quadruple inducible transgenic mouse governed by the podocin promoter, which in turn acts on the* mT*/*mG* transgene targeted under one Rosa26 allele and on the iDTR transgene inserted in the other Rosa26 allele. To develop this model, Wanner et al. created two different transgenic lines, both governed by podocin promoter, but with two different reporter transgenes (iDTR in one line and* mT*/*mG* in the other one). When crossed, these lines gave rise to the quadruple transgenic model, with one Rosa26 allele leading to iDTR expression and with the other Rosa26 allele carrying the* mT*/*mG* transgene (Figure 3(b)). This strategy enabled tagging of podocytes with GFP, while inducing iDTR expression and thus rendering the cells susceptible to toxin-induced ablation. By this it was possible to visualize and quantify by flow cytometry* de novo* generated podocytes, since they can only be red-colored due to the withdrawal of Doxycycline before the onset of the damage. This transgenic route presents intrinsic limits since it is not possible to identify the source of novel podocytes or to individuate progenitor population origin [52]. Intriguingly, a month after DT injection, the authors reported that about 38% of lost podocytes are replaced by newly generated Tomato Red^+^ podocytes. On the contrary, neither in the aging nephropathy model nor in the unilateral nephrectomy setting, an increase in Tomato Red^+^ podocytes was evidenced [52]. Data on the aging model have been already discussed above. Similar consideration can be made to explain why podocyte regeneration observed in the iDTR model cannot however be highlighted in the unilateral nephrectomy model, in which the major compensatory mechanism to the nephron loss is the hypertrophy of the survived glomeruli [52, 56]. However, even in the 5/6 nephrectomy and 5/6 nephrectomy + DOCA salt, Berger et al. did not observe podocyte regeneration [56].

In conclusion, from the available experimental data on transgenic mouse models, we can state that cells located in Bowman's capsule give rise to podocytes during the first weeks of age.

The role of PECs or other podocyte progenitors following injury is however still a matter of debate, with discrepancies among studies even on the possibility that podocyte regeneration may occur at all. What account for these discrepancies? One explanation is related to the fact that the renal progenitor cell activity may be more or less marked depending on the nature of injury, as described in liver stem cells [60], or depending on the magnitude of the damage. It is well known that elevated albuminuria impairs PEC differentiation into podocytes, as demonstrated by Peired et al. [72]. It is thus possible that podocyte regeneration could be visualized only in models in which regression of glomerular damage occurs. To this aim, specific podocyte ablation models are more suitable because the degree of podocyte loss could be more finely modulated [48]. In agreement with this hypothesis is the finding that, using the iDTR model, Wanner et al. provide evidence for podocyte regeneration when animals received a DT dose sufficient to cause a net loss of approximately 12% of all podocytes but without imposing any persistent gross damage to the tissue [52].

**Figure 4:**
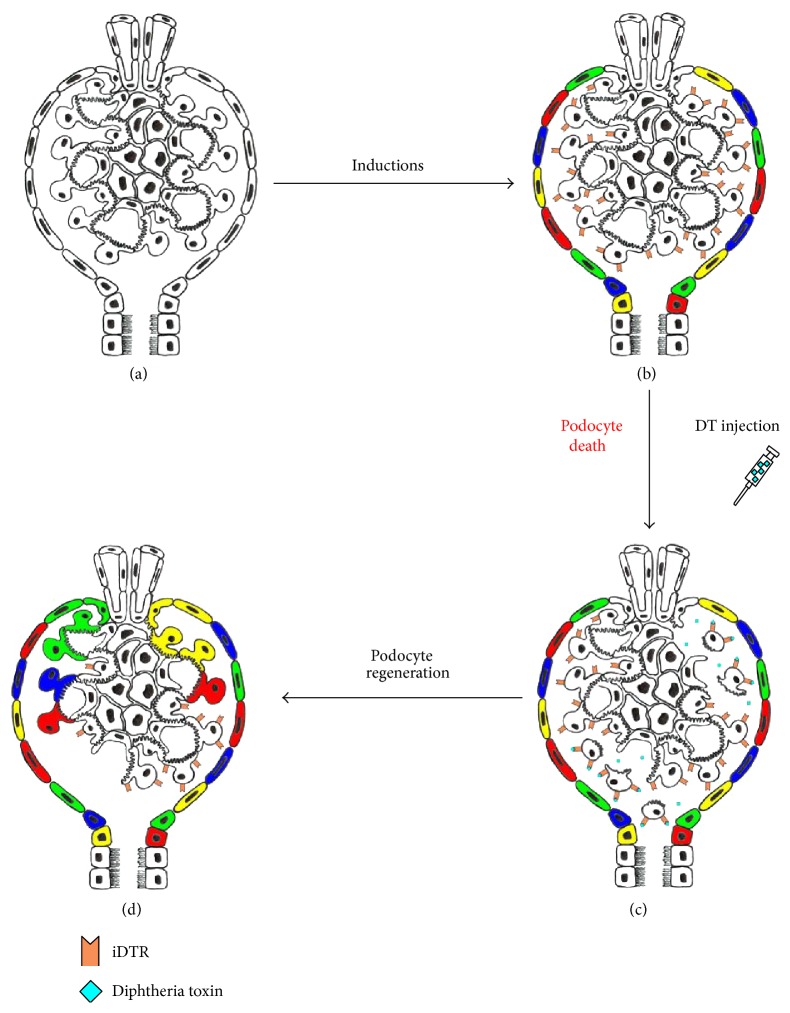
Hypothetical scheme of a combinatorial solution for PEC tagging and podocyte-specific ablation. (a) Glomerulus before induction of the two recombinatorial systems (Cre-LoxP and Flp-FRT), which can be induced independently and at different time points. (b) Once induced, the glomerulus would appear with Confetti-tagged PECs and iDTR-expressing podocytes. (c) At the prespecified time and dose, the operator could specifically ablate only podocytes. (d)* De novo* podocyte generation by the PEC compartment.

In addition, all the studies reviewed here evidence the necessity to find a PEC-specific promoter physiologically expressed only in this cell type. This will pave the way for further evolution of transgenic tactics finalized to a deep comprehension of the dynamics between PECs and podocytes. In this context, an important aim would be the creation of a multiple combinatorial inducible transgenic model in which both PECs and podocytes are engineered, the first with a fluorescent reporter and the second with a construct for cell ablation. A possible solution, for instance, could be the development of a Confetti-marked PECs, controlled by the Cre-LoxP system, combined to podocyte-targeted iDTR transgene expression controlled by the flippase recombinase that recognizes flippase recognition target (FRT) (FLp-FRT system) (Figure 4). The creation of a hypothetical transgenic model like this would require great efforts, but similar combinatorial tactics are actually under development in various organisms and organ system and hold great promises for regenerative studies [73, 74].

## 5. Conclusions

Transgenic animal models, recombinatorial strategies, and related lineage tracing analysis are powerful tools that can be widely applied to understand the mechanisms that govern kidney dynamics and regeneration. Transgenic approaches are not only changing our views on kidney regeneration capabilities but also opening to researchers new perspectives in various aspects of kidney physiology that will grant great advantages in understanding this complex organ. Notwithstanding, transgenic-related techniques still need a further development in order to unequivocally identify and characterize stem and progenitor populations involved in podocyte replacement.
